# Notes and News

**Published:** 1891-12-19

**Authors:** 


### Notes and News.

COLLECTED AND COMMUNICATED.

At a meeting of the Hospitals Association on November 25, at the Royal Free Hospital, Gray's Inn Road, Mr. Conrad W. Thies read a paper on "A Model Hospital"—the new general hospital, at Hamburg—and Mr. Jonathan Hutchinson, F.R.S., the Chairman, said he thoroughly endorsed the high commendation Mr. Thies had passed on this great institution. Every precaution had been taken to prevent anything like hospital contagion and to ensure the very highest degree of atmospheric purity and freedom from germ contamination.

Mr. Solley, F.R.C.S., in criticising the establishment of a large hospital outside London, on the principle of the Hamburg institution, pointed out the difficulties connected with chemical instruction, and that it would strongly militate against securing the services of the leading medical men, whose other duties would in many cases preclude their taking a long railway journey to deliver a lecture at an institution situated outside the metropolis.

We received a post-card last week, headed "Myxœdema," and signed "M.B., B. Sc., Lond. Univ." The card contains but six lines, and yet in these six lines it succeeds in being ungrammatical, illogical, and impertinent. We do not wish to make "M. B., B. Sc. Lond. Univ." blush for himself; and therefore we do not publish his post-card.

A quarterly General Court of the Governors of the Middlesex Hospital was held on Thursday, 26th ult. Mr. Bell Sedgwick, J.P., occupied the chair, and there was a numerous attendance. The Court unanimously appointed Dr. James Kingston Fowler to the office of fourth physician, in place of Dr. David W. Finley, resigned, and gave authority for the sale of £3,191 Consolidated Stock to repay a loan from the bankers and to meet current expenses. It was also decided to authorise the necessary expenditure for the installation of the electric light in certain portions of the Hospital and the Trained Nurses' Institute, Nurses' Home and Medical School.

The Secretary of the Hospital for Consumption at Ventnor appeals on behalf of the Institution, which has now for twenty-two years done much to alleviate the sufferings inseparable from this sad complaint. During that period no less than 10,000 patients have passed through the Hospital, which has been, from time to time, enlarged by the liberality of individual donors of houses till it now affords accommodation for 135 patients, each of whom has a separate bed-room, and of whom only groups of four or six are required to use the sitting-rooms in common. By these means, aided by the effective ventilation which the Board, availing themselves of the most recent sanitary experience, have applied to every part of the building, they are enabled to give every patient an abundant supply of pure air. The results have been most encouraging, the greater number of the patients deriving decided benefit from their stay at Ventnor. The spiritual needs of the patients are carefully attended to, a Church of England chaplain being in constant attendance, and free access to inmates of any religious denomination being given to ministers whom they may wish to see. The buildings include a commodious and well-ventilated chapel, erected by private benevolence. Frequent entertainments are given throughout the winter months, and the beautiful grounds afford exercise and pleasure to those patients able to avail themselves of it whenever the weather permits. The annual cost of maintaining the Institution in efficiency exceeds £9,000, whilst the annual subscriptions yield only £1,800. The Board of Management appeals specially for an increase of annual subscribers, but donations will be thankfully received.

